# Epigenetic Dysregulation of the NKX2‐1/SPDEF Axis Drives Persistent Goblet Cell Differentiation and Epithelial Barrier Dysfunction in Chronic Obstructive Pulmonary Disease

**DOI:** 10.1002/resp.70201

**Published:** 2026-01-20

**Authors:** Ayaka Shiota, Keiko Kan‐o, Yumiko Ishii, Tomoaki Koga, Takeshi Sawada, Kei‐ichiro Yasunaga, Shingo Usuki, Tatsuya Katsuno, Shigesato Inoue, Tomohiro Ogawa, Akihiro Jo, Satoru Fukuyama, Mitsuyoshi Nakao, Hiroaki Ogata, Mizuho A. Kido, Sachiko Tsukita, Koichiro Matsumoto, Isamu Okamoto

**Affiliations:** ^1^ Department of Respiratory Medicine, Graduate School of Medical Sciences Kyushu University Fukuoka Japan; ^2^ Division of Respiratory Medicine National Hospital Organization Fukuoka National Hospital Fukuoka Japan; ^3^ Department of Respiratory Medicine Tokyo Women's Medical University Tokyo Japan; ^4^ Division of Histology and Neuroanatomy, Department of Anatomy and Physiology, Faculty of Medicine Saga University Saga Japan; ^5^ Department of Medical Cell Biology Institute of Molecular Embryology and Genetics, Kumamoto University Kumamoto Japan; ^6^ Liaison Laboratory Research Promotion Center IMEG, Kumamoto University Kumamoto Japan; ^7^ Center for Anatomical Studies, Graduate School of Medicine Kyoto University Kyoto Japan; ^8^ KOKORO‐Biology Group, Graduate School of Frontier Biosciences Osaka University Osaka Japan; ^9^ Department of Respiratory Medicine National Hospital Organization Omuta National Hospital Omuta Japan; ^10^ Advanced Comprehensive Research Organization Teikyo University Tokyo Japan; ^11^ Laboratory of Barriology and Cell Biology, Graduate School of Frontier Biosciences Osaka University Osaka Japan; ^12^ Department of Medicine, Division of Oral and Medical Management Fukuoka Dental College Fukuoka Japan

**Keywords:** airway epithelial barrier function, chronic obstructive pulmonary disease, epigenetics, goblet cells, single‐cell analysis

## Abstract

**Background and Objective:**

Despite improved respiratory symptoms after smoking cessation, patients with chronic obstructive pulmonary disease (COPD) remain susceptible to exacerbations and persistent airway inflammation, wherein the underlying mechanisms for sustained inflammation remain unclear. To address this knowledge gap, we investigated the persistence of airway epithelial barrier dysfunction in ex‐smokers with COPD and examined the relationship between goblet cell hyperplasia and barrier dysfunction.

**Methods:**

We analysed differentiated primary bronchial epithelial cells from never smokers with normal lung function, ex‐smokers (> 10‐year cessation), and current smokers with COPD using RNA sequencing, ATAC sequencing, and single‐cell analyses to examine barrier function and cell differentiation.

**Results:**

Genes associated with ciliary formation and motility were progressively downregulated from never smokers to ex‐smokers to current smokers with COPD. The expression of junction‐associated molecules was decreased in both ex‐smokers and current smokers, showing a significant inverse correlation with the proportion of MUC5AC‐positive cells. Single‐cell analyses revealed distinct alterations in cell differentiation trajectories, particularly persistent goblet cell hyperplasia associated with increased expression of the transcription factor *SPDEF*, linked to epigenetic changes in the *NKX2‐1* gene regulatory regions.

**Conclusion:**

Epigenetic mechanisms maintain persistent alterations in airway epithelial differentiation after smoking cessation, leading to mucus‐producing cell hyperplasia through dysregulation of the NKX2‐1/SPDEF axis. This hyperplasia correlates with reduced junction‐associated molecule expression and subsequent barrier dysfunction. Therapeutic strategies targeting epithelial barrier restoration and/or normalisation of epigenetic dysregulation may benefit patients with COPD, even after smoking cessation.

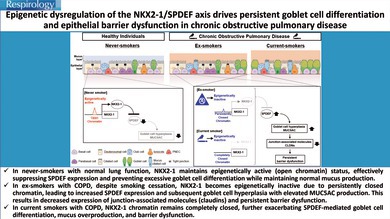

## Graphical abstract

This study investigated airway epithelial barrier dysfunction persistence in ex‐smokers with COPD and underlying mechanisms using single‐cell RNA and ATAC sequencing. Epigenetic NKX2‐1/SPDEF dysregulation maintains goblet cell hyperplasia and barrier dysfunction despite long‐term smoking cessation. The findings may explain continued exacerbation susceptibility and suggest potential epigenetic therapies for COPD.

**See related** editorial

## 1 Introduction

Environmental exposure‐related impairment of airway epithelial barriers mediates the pathogenesis of various chronic noncommunicable diseases, including chronic obstructive pulmonary disease (COPD) and asthma [1, 2, 3]. Barrier dysfunction enables pathogen colonisation and alterations, triggering immune responses against environmental substances and leading to chronic tissue inflammation and barrier impairment.

Epithelial barrier integrity in the airways is maintained through two key mechanisms: mucociliary clearance and intercellular junctional complexes. These complexes comprise apical tight junctions and underlying adherens junctions between adjacent epithelial cells [4, 5]. Cigarette smoke extracts (CSE) compromise epithelial barrier function in vitro by downregulating junction‐associated proteins, including claudins, occludin, E‐cadherin, and zonula occludens‐1 [6, 7, 8, 9]. In patients with COPD, compared to healthy controls, the bronchial epithelium and lung tissues exhibit reduced expression of junction‐associated proteins that potentially facilitate enhanced penetration of inhaled substances and excessive inflammation [10, 11, 12]. Although there is evidence of ongoing airway inflammation and exacerbations after smoking cessation, it remains unclear whether these barrier abnormalities persist in ex‐smokers with COPD.

Cigarette smoke exposure promotes goblet cell hyperplasia and upregulates MUC5AC expression through epidermal growth factor receptor signalling, and thereby contributes to airway obstruction [13, 14]. Notably, a recent longitudinal study revealed that, compared to healthy individuals, ex‐smokers with COPD had elevated sputum MUC5AC levels with high baseline MUC5AC concentrations, but not MUC5B, which correlated with an increased frequency of exacerbation [15]. Thus, persistent goblet cell hyperplasia may compromise both mucociliary clearance and epithelial cell‐to‐cell contact to potentially perpetuate barrier dysfunction and mucosal inflammation. The differentiation of airway epithelial cells into goblet cells is primarily promoted by the transcription factor SAM‐pointed domain‐containing Ets‐like factor (SPDEF), while NK2 homeobox 1 (NKX2‐1), an upstream transcription factor of SPDEF, downregulates SPDEF expression, suggesting a critical regulatory axis controlling mucus production [16, 17]. However, the pathomechanisms underlying the potential persistence of goblet cell hyperplasia and barrier dysfunction in COPD remain unexplored.

Using differentiated bronchial epithelial cells from never‐smokers with normal lung function, ex‐smokers, and current smokers with COPD, we investigated the persistence of airway epithelial barrier dysfunction in patients with COPD who quit smoking for at least 10 years. Furthermore, we examined the relationship between goblet cell hyperplasia and barrier dysfunction and investigated whether these alterations were maintained through epigenomic and transcriptomic regulatory mechanisms in patients with COPD.

## 2 Methods

### 2.1 Culture of Primary Bronchial Epithelial Cells

Primary bronchial epithelial cells (PBECs) were collected and cultured as described previously [18, 19]. PBECs were obtained during routine fiberoptic bronchoscopy by bronchial brushing from the same anatomical region (bronchial generations 4–7) in lobes without pulmonary nodules. The patients were classified as never smokers with normal lung function (pack‐years ≤ 3 and at least 20 years of smoking cessation), ex‐smokers with COPD (at least 10 years of smoking cessation), or current smokers with COPD. All patients with COPD were diagnosed according to the Global Initiative for Chronic Obstructive Lung Disease guidelines and had emphysema on chest computed tomography. Table S1 shows demographic data of patients from whom the cells used in the corresponding experiments in each figure were derived.

The study was conducted in accordance with the Declaration of Helsinki. The study protocol was approved by the Kyushu University Institutional Review Board for Clinical Research (2023‐44), and all participants provided written informed consent.

Air‐liquid interface (ALI) culture conditions are described in the Supporting Information Methods.

### 2.2 Electron Microscopy

Ultrastructural analysis was performed by transmission electron microscopy (TEM) and backscattered electron scanning electron microscopy (BSE‐SEM). The specimens were fixed, post‐fixed, and processed according to the standard protocols described in the Supporting Information Methods.

### 2.3 RNA Sequencing

Total RNA from PBECs cultured simultaneously under ALI conditions from never smokers with normal lung function (*n* = 3), ex‐smokers with COPD (*n* = 2), and current smokers with COPD (*n* = 3) was extracted and sequenced on a NextSeq 500 sequencer with 75‐bp single‐end reads. The detailed library preparation and analysis protocols are provided in the Supporting Information Methods.

### 2.4 Assay for Transposase‐Accessible Chromatin via Sequencing

ALI‐PBECs from never smokers with normal lung function (*n* = 3), ex‐smokers with COPD (*n* = 2), and current smokers with COPD (*n* = 3) were dissociated into single cells and subjected to transposase‐Accessible Chromatin via Sequencing (ATAC‐seq) using an ATAC‐seq kit (Active Motif, #53150) according to the manufacturer's instructions [20]. The analysis protocols are described in the Supporting Information Methods.

### 2.5 Quantitative Reverse‐Transcription PCR

Using quantitative reverse‐transcription PCR (qRT‐PCR), total RNA was isolated from ALI‐PBECs using TRI Reagent (Molecular Research Centre Inc., Cincinnati, OH, USA) and processed following the standard protocols detailed in the Supporting Information. Primer sequences are provided in Table S2.

### 2.6 Immunofluorescence Staining

The differentiated cells were fixed, blocked, and incubated with primary antibodies, and thereafter incubated with fluorescence‐conjugated secondary antibodies. Images were captured using confocal laser scanning microscopes: Andor BC43 Benchtop confocal microscope (Andor, Belfast, UK); LSM700 or LSM800 equipped with Airyscan (Carl Zeiss, Oberkochen, Germany). Detailed protocols, quantitative analyses of fluorescence intensity, and antibody information are provided in the Supporting Information Methods.

### 2.7 Preparation and Treatment of CSE

CSE was prepared as previously described [6, 8] and 100% CSE preparation has been described in the Supporting Information Methods. Briefly, 200 μL culture medium (control) or 10% CSE diluted with culture medium was added to the apical chamber and changed every 12 h. Cells were maintained with 500 μL culture medium without CSE in the basal chamber, and the medium was changed every 24 h. In the constant‐exposure model, exposure to 10% CSE was sustained for 144 h whereas in the temporary‐exposure model, exposure to 10% CSE was conducted for the first 44 h and then switched to a control medium, which was changed every 12 h for 144 h after CSE exposure.

### 2.8 Measurement of Transepithelial Electrical Resistance

Transepithelial electrical resistance (TEER) was evaluated using a Millicell‐ERS 2 V‐Ohm metre (Millipore Co., Bedford, MA) in accordance with the standard protocols presented in the Supporting Information Methods.

### 2.9 Single‐Cell RNA Sequencing

For single‐cell RNA sequencing (scRNA‐seq), PBECs from a never smoker with normal lung function (*n* = 1), ex‐smokers with COPD (*n* = 2), and a current smoker with COPD (*n* = 1) were cultured simultaneously under ALI conditions for more than 4 weeks. Monolayer formation and epithelial cell polarisation were monitored using TEER measurement. Five thousand single cells were subjected to a Chromium Controller (10× Genomics, Pleasanton, CA, USA). The library was constructed with the Chromium Single Cell 3′ v3.1 kit (10× Genomics) and sequenced using an Illumina NovaSeq X Plus. Twenty‐one clusters were visualised using specific marker genes. The analysis protocols are detailed in the Supporting Information Methods.

### 2.10 Single‐Cell ATAC Sequencing

For single‐cell ATAC sequencing (scATAC‐seq), we used single ALI‐PBECs cultured simultaneously with those prepared for scRNA‐seq as described above. Single cells were washed twice with PBS (−) and resuspended in 0.04% BSA/PBS. Nuclei were isolated and 5000 nuclei were subjected to library preparation. The library was constructed using the Chromium Next GEM Single Cell ATAC kit v2 (10× Genomics) and sequenced using an Illumina NovaSeq X Plus. The analysis protocols are described in the Supporting Information Methods.

### 2.11 Statistical Analysis

Data were expressed as the mean ± standard error (SEM) unless stated otherwise. Groups were compared using one‐way or two‐way analysis of variance (ANOVA) followed by Tukey's multiple comparison test. Correlations were examined using the Spearman's correlation. All statistical analyses were conducted using the GraphPad Prism 10.2 software (GraphPad Software, San Francisco, CA, USA). Differences were considered statistically significant at *p* < 0.05.

## 3 Results

### 3.1 Worsening Mucociliary Clearance in the Airway Epithelium of Ex‐ and Current Smokers With COPD

**FIGURE 1:**
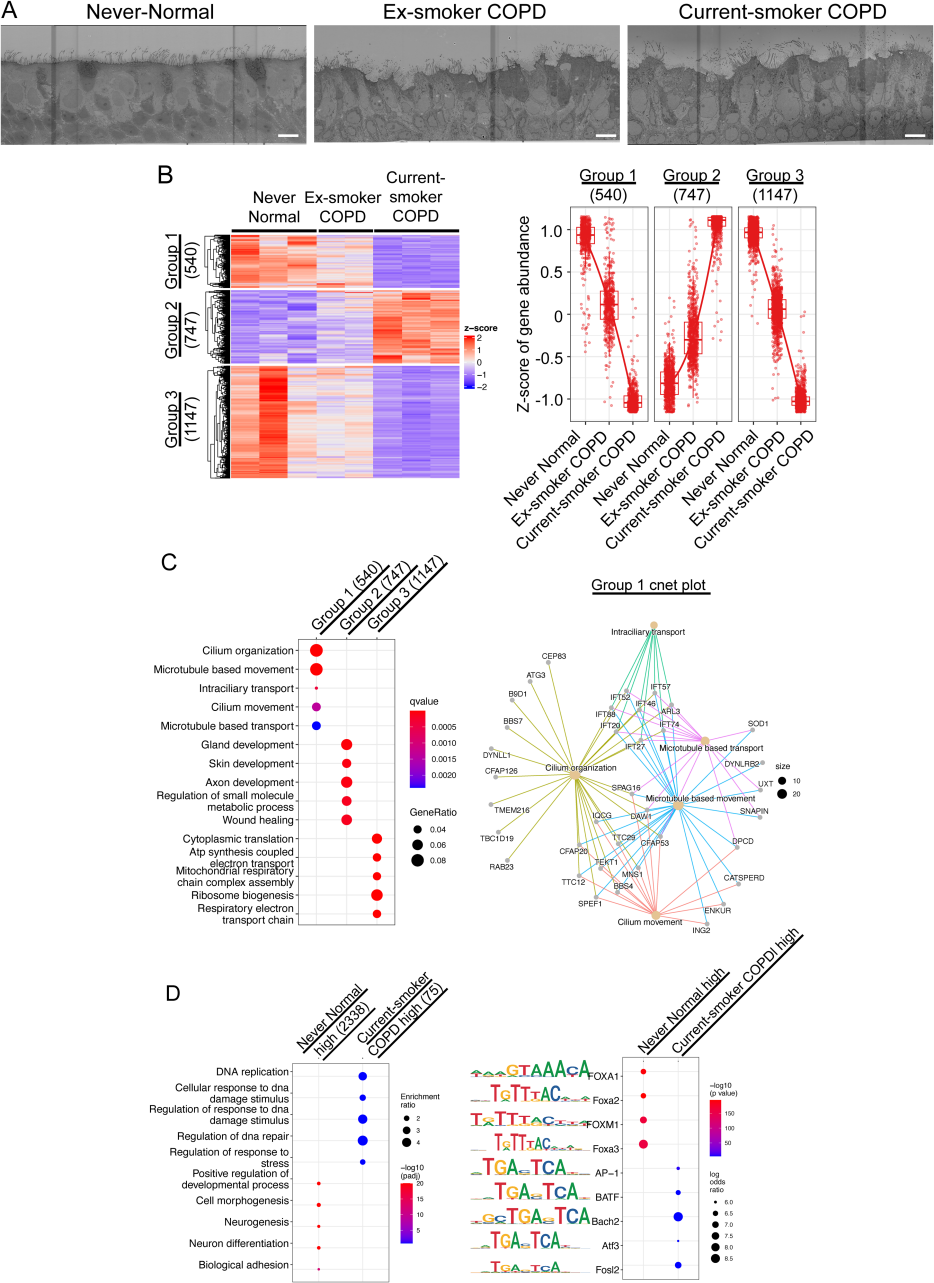
Bulk RNA and ATAC sequencing analysis of differentiated primary bronchial epithelial cells from healthy never‐smokers (*n* = 3), ex‐smokers (*n* = 2), and current smokers (*n* = 3) with COPD. (A) Comparative ultrastructural analysis of airway epithelium by transmission electron microscopy. Scale bar, 10 μm. (B) Hierarchical *k*‐means clustering analysis revealing transcriptional alterations in COPD airway epithelium compared to never‐smokers with normal lung function shown in a heatmap (left panel) and dot plot (right panel). (C) Cluster‐specific gene ontology enrichment analysis revealing distinct biological signatures (left panel) and biological pathway mapping of genes associated with the Cluster 1 signature (right panel). (D) Cluster‐specific gene ontology enrichment analysis revealing distinct biological signatures (left panel). Motif enrichment analysis by Homer comparing never‐smokers with normal lung function and current smokers with COPD (right panel) [Correction added on 24 February 2026, after first online publication: Figure 1 has been corrected in this version.].

Under ALI conditions for > 21 days, we simultaneously differentiated PBECs from never smokers with normal lung function and current and ex‐smokers with COPD. On comparative electron microscopy analysis, control samples from never smokers with normal lung function exhibited uniformly aligned ciliated epithelium (Figure 1A). In contrast, samples from ex‐ and current smokers with COPD showed ciliated epithelial cells replaced by secretory granule‐containing goblet cells, resulting in irregular apical surfaces (Figure 1A). Bulk RNA sequencing analysis of the differentiated airway epithelium revealed distinct transcriptome profiles among samples (Figure S1A). Differentially expressed gene (DEG) analysis identified three distinct clusters. Group 1 (540 DEGs) and Group 3 (1147 DEGs) showed progressively decreased gene expression from never smokers to ex‐smokers with COPD to current smokers with COPD, whereas Group 2 (747 DEGs) demonstrated progressively increasing expression in the same order (Figure 1B). Gene ontologic analysis showed that Group 1 contained genes associated with cilia formation and motility as well as microtubule‐based movement and transport pathways (Figure 1C). Compared with never smokers with normal lung function, the expression of these genes progressively decreased in ex‐smokers followed by current smokers with COPD (Figure S1B). Gene set enrichment analysis (GSEA) showed dysregulation of cell junction organisation‐related gene expression in the current smoker with COPD, compared with never smokers with normal lung function (Figure S2A). To understand genome‐wide epigenetic status among samples, we performed ATAC‐seq analysis and found that 2338 and 75 differentially accessible regions (DARs) were highly accessible in never smokers with normal lung function and current smokers with COPD samples (Figure S2B). Pathway analysis showed that DARs in the never smokers with normal lung function high group were related to the positive regulation of developmental processes, cell morphogenesis, and biological adhesion (Figure 1D, left panel), whereas DARs in the current smokers with COPD high group were associated with DNA repair and stress responses. Interestingly, motif analyses showed that the binding motif of the Forkhead box (FOX) family transcription factor that maintains epithelial cell barrier integrity in respiratory epithelial cells, a critical upstream transcription factor of SPDEF [21], and the binding motif of AP‐1 transcription factor, which is important for airway inflammation and remodelling [22], were enriched in never smokers with normal lung function high group and current smokers with COPD high group, respectively (Figure 1D, right panel). These data suggest that, at both the transcriptomic and epigenomic levels, the mucociliary epithelial barrier system is dysregulated in ALI cultured‐PBECs from ex‐smokers and current smokers with COPD.

### 3.2 Decreased Gene and Protein Expression of Junction‐Associated Molecules in Airways of Current and Ex‐Smokers With COPD

**FIGURE 2:**
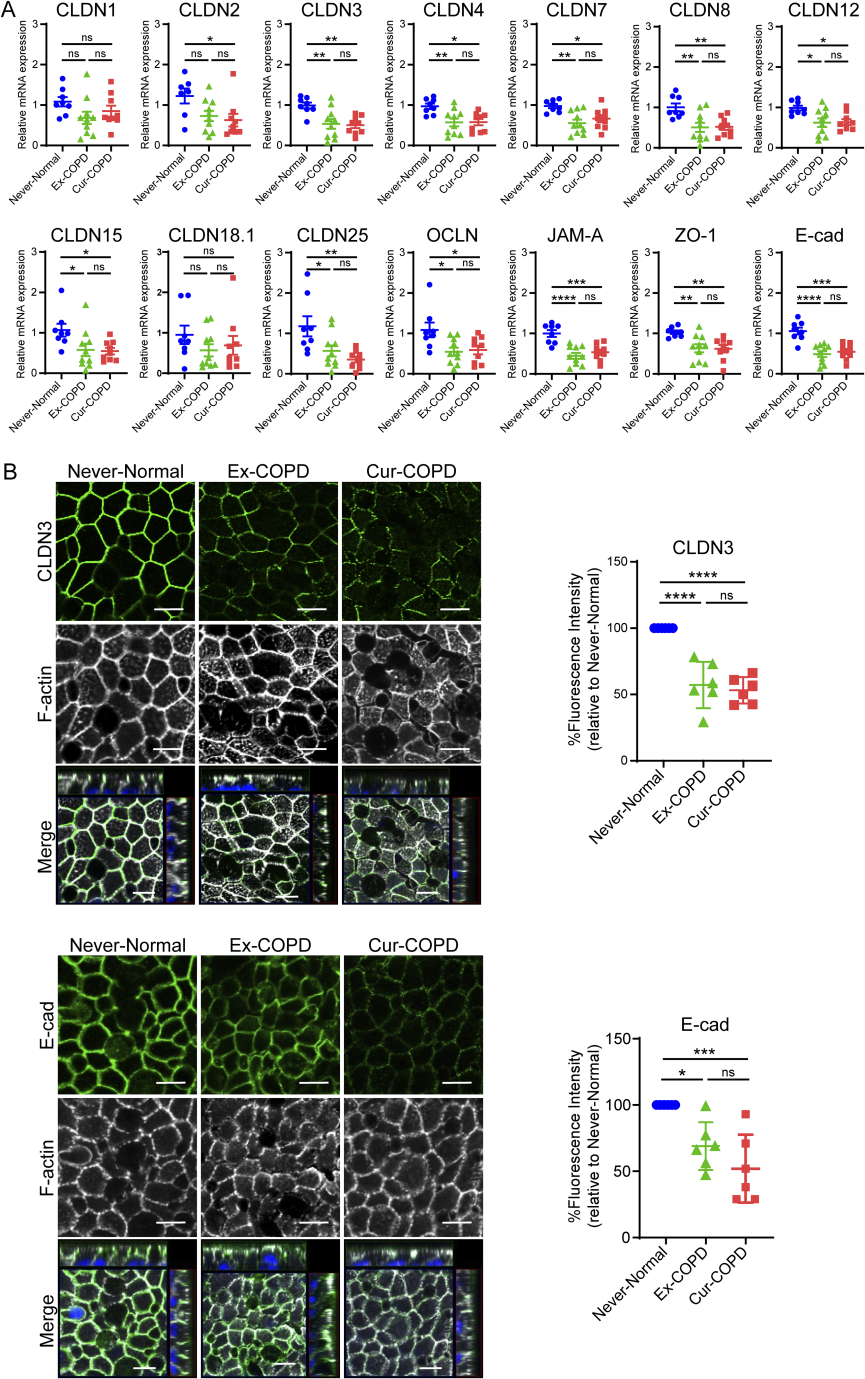
Comparative gene expression and immunofluorescence analysis of junction‐associated molecules in differentiated primary bronchial epithelial cells from healthy never‐smokers, ex‐smokers, and current smokers with COPD. (A) Gene expression was analysed by real‐time PCR and normalised to that of 18S rRNA. Data represent means ± SEM (*n* = 7–10 independent donors per group). (B) Confocal immunofluorescence microscopy of claudin‐3 and E‐cadherin (green), F‐actin (white), and DAPI staining of nuclei (blue). Scale bar, 10 μm. Images are representative of six independent experiments (*n* = 4–6 independent donors per group). Quantification of fluorescence intensity normalised to never‐smokers with normal lung function (set at 100%). Data represent the mean ± SEM (*n* = 4–6 independent donors per group). *p < 0.05, **p < 0.01, ***p < 0.001, ****p < 0.0001 by one‐way ANOVA *post hoc* with Tukey test. CLDN, claudin; E‐cad, E‐cadherin; JAM, junctional adhesion molecule; ns, not significant; OCLN, occludin; ZO, zonula occludens [Correction added on 24 February 2026, after first online publication: Figure 2 has been corrected in this version.].

On Day 21 of ALI culture, the differentiated airway epithelium of current smokers with COPD exhibited a significant TEER reduction compared to that of never smokers with normal lung function as well as ex‐smokers with COPD (Figure S3). Compared with never smokers with normal lung function, current and ex‐smokers with COPD had decreased expression of multiple genes encoding tight junction‐ and adherens junction‐associated molecules (Figure 2A). Immunofluorescence analysis of claudin‐3 and E‐cadherin protein expression revealed decreased levels in the airway epithelium of both current and ex‐smokers with COPD (Figure 2B), which were consistent with the gene expression findings.

### 3.3 Negative Impact of Goblet Cell Hyperplasia on the Expression of Junction‐Associated Molecules in the Airway Epithelium

**FIGURE 3:**
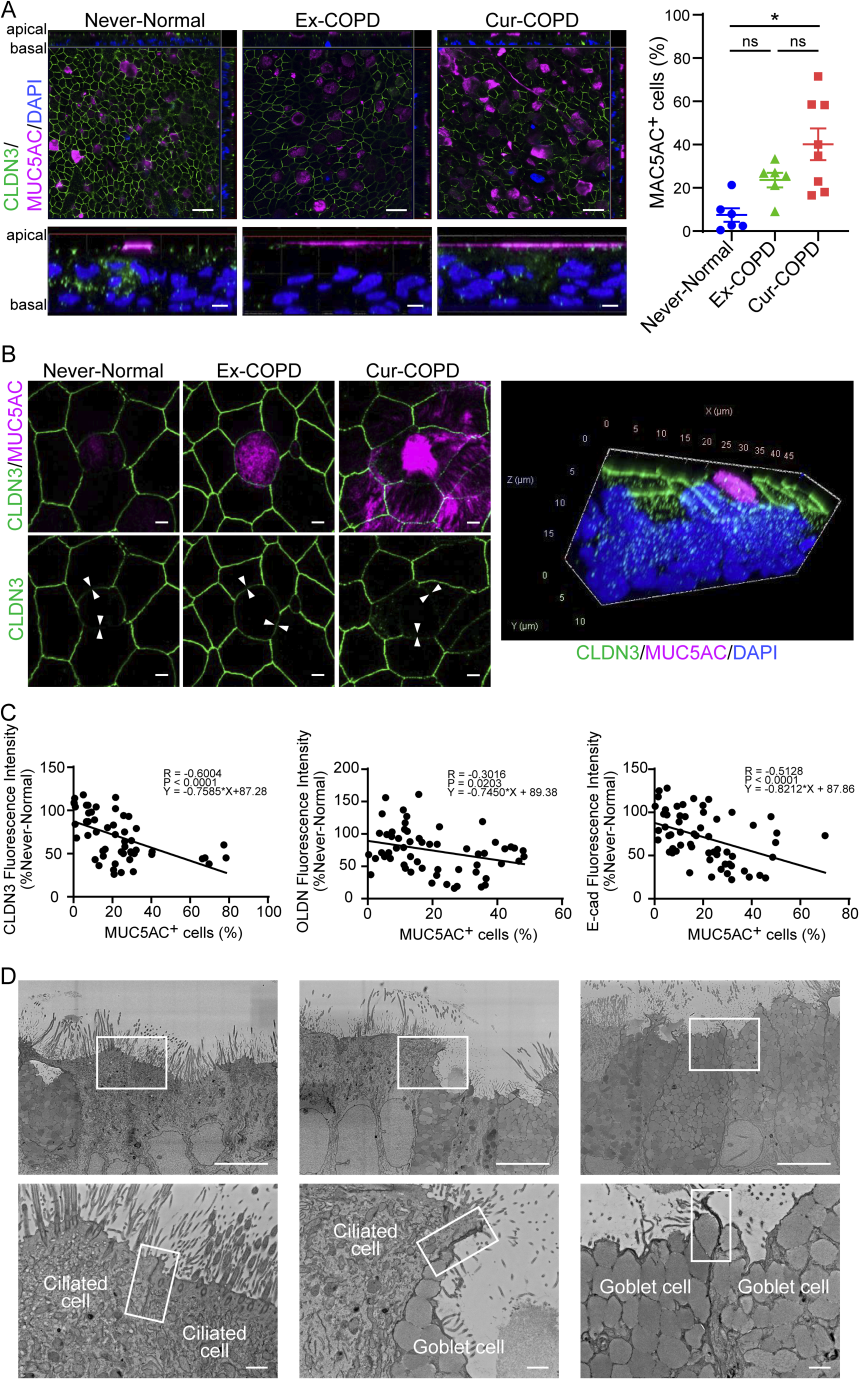
Characterisation of junction‐associated molecules expression between goblet cells and their adjacent epithelial cells. Confocal immunofluorescence microscopy of (A and B) claudin‐3 (green), MUC5AC (magenta), and DAPI staining of nuclei (blue), and (A) assessment of MUC5AC‐positive cell frequency. (A) Scale bar, 20 μm (upper panels) and 2 μm (lower panels). (B) Scale bar, 2 μm. Arrowheads mark the goblet cell‐adjacent cell boundaries. Images are representative of six independent experiments (*n* = 4–8 independent donors per group). (C) Correlation analysis of junction‐associated molecule expression intensity and proportion of MUC5AC‐positive cells. (D) Ultrastructural assessment of intercellular junctions using backscattered electron SEM. Scale bar, 10 μm (upper panels) and 1 μm (lower panels). Data represent the mean ± SEM (*n* = 4–8 independent donors per group). *p < 0.01, one‐way ANOVA *post hoc* with Tukey's test and Spearman's correlation, as appropriate. CLDN, claudin; E‐cad, E‐cadherin; ns, not significant; OCLN, occludin [Correction added on 24 February 2026, after first online publication: Figure 3 has been corrected in this version.].

In patients with COPD, immunofluorescence revealed goblet cell hyperplasia and MUC5AC‐positive mucus accumulation on the apical surface in both current and ex‐smokers (Figure 3A). Compared to never smokers with normal lung function, MUC5AC‐positive cells were increased in ex‐smokers and significantly elevated in current smokers with COPD (Figure 3A). The intercellular claudin‐3 labelling between goblet and adjacent ciliated cells was attenuated in the cells from patients with COPD and never smokers with normal lung function (Figure 3B). On combining data from never smokers with normal lung function as well as current and ex‐smokers with COPD, the proportion of MUC5AC‐positive cells negatively correlated with the expression of junction‐associated molecules (claudin‐3, occludin, and E‐cadherin; Figure 3C). Tight junctions were localised within approximately 1 μm from the apical surface. Ultrastructural evaluation using electron microscopy revealed robust membrane adhesion between neighbouring ciliated cells extending to the apical surface (Figure 3D, left panel). In contrast, observations of the interfaces between ciliated cells and adjacent goblet cells demonstrated structural deficits at the tight junction level, which coincided with the secretion of intracellular granules from goblet cells (Figure 3D, middle and right panels).

### 3.4 CSE‐Induced Goblet Cell Hyperplasia Compromises Epithelial Barrier Integrity

**FIGURE 4:**
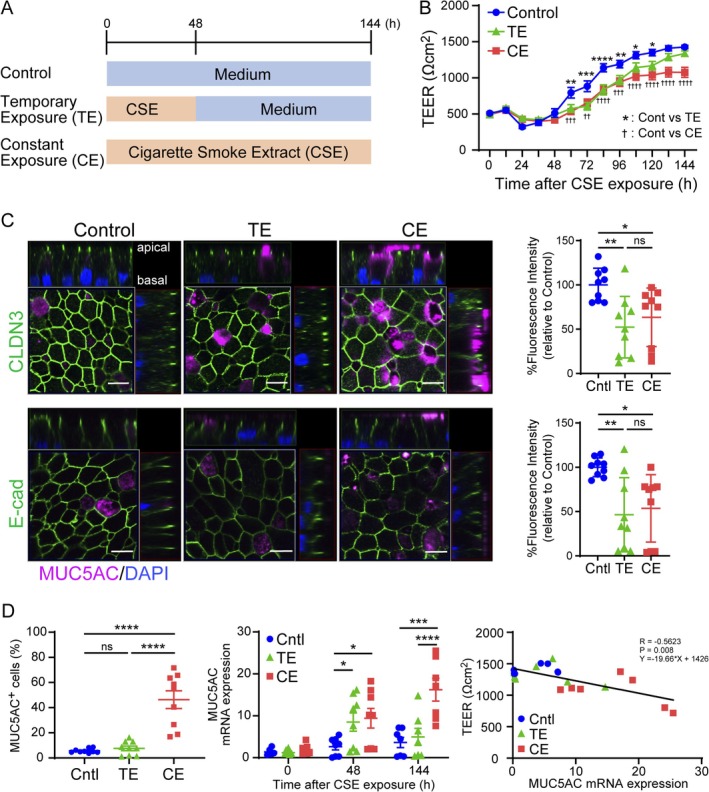
Effects of cigarette smoke extract (CSE) exposure on epithelial barrier integrity, goblet cells, and junction‐associated molecule expression in differentiated primary bronchial epithelial cells. (A) Experimental design for CSE exposure in differentiated airway epithelial cells derived from healthy never‐smokers with normal lung function. (B) Time‐course analysis of transepithelial electrical resistance (TEER). (C) Confocal immunofluorescence microscopy of claudin‐3 (green), MUC5AC (magenta), and DAPI staining of nuclei (blue) and quantifying fluorescence intensity. Scale bar, 10 μm. Images are representative of three independent experiments. (D) Assessment of MUC5AC‐positive cell frequency (left panel). Quantitative analysis of MUC5AC gene expression levels using real‐time PCR (middle panel). Correlation between TEER values and MUC5AC gene expression levels (right panel). Data represent the mean ± SEM (*n* = 3 independent donors with three replicates per group). *p < 0.05, **p < 0.01, ***p < 0.001, ^††^ *p* < 0.01, ^†††^ *p* < 0.001, ^††††^ *p* < 0.0001 by one‐ or two‐way ANOVA *post hoc* with Tukey test and Spearman correlation as appropriate. CLDN, claudin; E‐cad, E‐cadherin; ns, not significant [Correction added on 24 February 2026, after first online publication: Figure 4 has been corrected in this version.].

To investigate whether goblet cell hyperplasia and downregulation of junction‐associated molecules in the airway epithelium in COPD were attributable to CSE, we conducted both temporary‐ and constant‐exposure experiments using CSE on the airway epithelium obtained from never smokers with normal lung function (Figure 4A). At 60 h after initiating CSE stimulation, both the temporary‐ and constant‐exposure groups exhibited decreased TEER compared to the control group. Although the constant‐exposure group showed a progressive widening of TEER differences relative to the control over time, the temporary‐exposure group demonstrated TEER recovery to levels comparable to those of the control group (Figure 4B). Immunofluorescence analysis conducted 144 h after CSE stimulation revealed decreased expression of claudin‐3 and E‐cadherin in both the temporary‐ and constant‐exposure groups (Figure 4C) whereas the proportion of MUC5AC‐positive cells increased only in the constant exposure group (Figure 4C,D, left panel). This dissociation suggests that barrier integrity, as measured by TEER and goblet cell hyperplasia as assessed by MUC5AC expression, may recover more rapidly than the normalisation of tight junction protein expression, indicating distinct temporal dynamics in epithelial repair mechanisms. Compared to controls, both the temporary‐ and constant‐exposure groups showed elevated MUC5AC gene expression levels 48 h after CSE stimulation (Figure 4D, middle panel). However, at 144 h, the temporary‐exposure group recovered to control levels, whereas the constant‐exposure group maintained an elevated expression (Figure 4D, middle panel). MUC5AC gene expression levels 144 h after CSE stimulation showed a significant negative correlation with TEER values (Figure 4D, right panel).

### 3.5 Increased Proportion of MUC5AC‐Positive Mucous Ciliated Cells and Goblet Cells in Ex‐Smokers and Current Smokers With COPD

**FIGURE 5:**
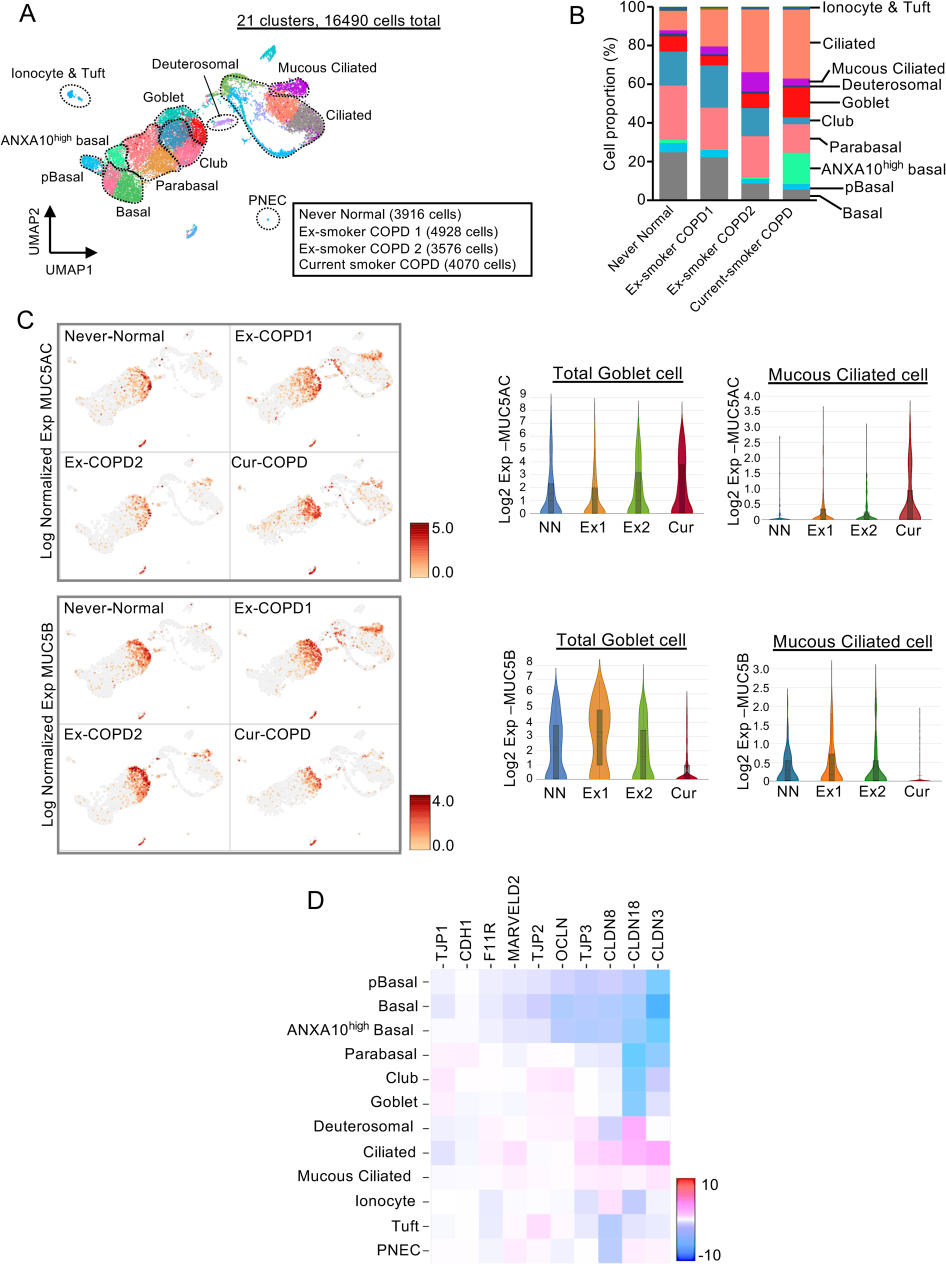
Single‐cell RNA sequencing analysis comparing differentiated primary airway epithelial cells from a healthy never smoker, two ex‐smokers, and a current smoker with COPD. (A) Uniform manifold approximation and projection (UMAP) visualisation and cell cluster identification of scRNA‐seq dataset. (B) Relative proportions of cell populations among groups (right panel). (C) MUC5AC and MUC5B gene expression visualised by UMAP, and violin plots comparing their expression levels in goblet cells and mucous ciliated cells across groups. (D) Cell cluster‐based heatmap analysis of junction‐associated molecule gene expression [Correction added on 24 February 2026, after first online publication: Figure 5 has been corrected in this version.].

Furthermore, scRNA‐seq analysis of ALI‐cultured PBECs from never smokers with normal lung function and current and ex‐smokers with COPD identified 21 distinct epithelial cell clusters, including basal, parabasal, club, goblet, deuterosomal, and ciliated cells (Figure 5A). Quantitative assessment of cell‐cluster distributions showed that, compared to never smokers with normal lung function, ex‐smokers with COPD showed an increase in mucous ciliated cells and a decrease in basal cells (Figure 5B). In contrast, current smokers with COPD exhibited an increased proportion of goblet and basal cells with high Annexin A10 expression and concurrent reductions in club and basal cells (Figure 5B). MUC5AC gene expression was observed in mucous ciliated cells, which showed an increased proportion in ex‐smokers with COPD, whereas current smokers with COPD showed expression in both mucous ciliated and goblet cells (Figure 5C). On comparison of the MUC5AC and MUC5B gene expression levels in goblet and mucous ciliated cells, MUC5AC expression was elevated in current smokers with COPD, whereas MUC5B expression decreased in current smokers with COPD (Figure 5C). The MUC5AC/MUC5B gene expression ratio (average) in goblet cells was 4.06 in the current smokers with COPD, 1.17 in the never smokers with normal lung function, and 0.74 in ex‐smokers with COPD. When examining a heatmap of the gene expression of junction‐associated molecules across clusters, ciliated cells showed the highest expression, whereas mucous ciliated and goblet cells showed lower expression (Figure 5D).

### 3.6 Epigenetic Alterations Drive Aberrant Goblet Cell Differentiation in COPD Airways

**FIGURE 6:**
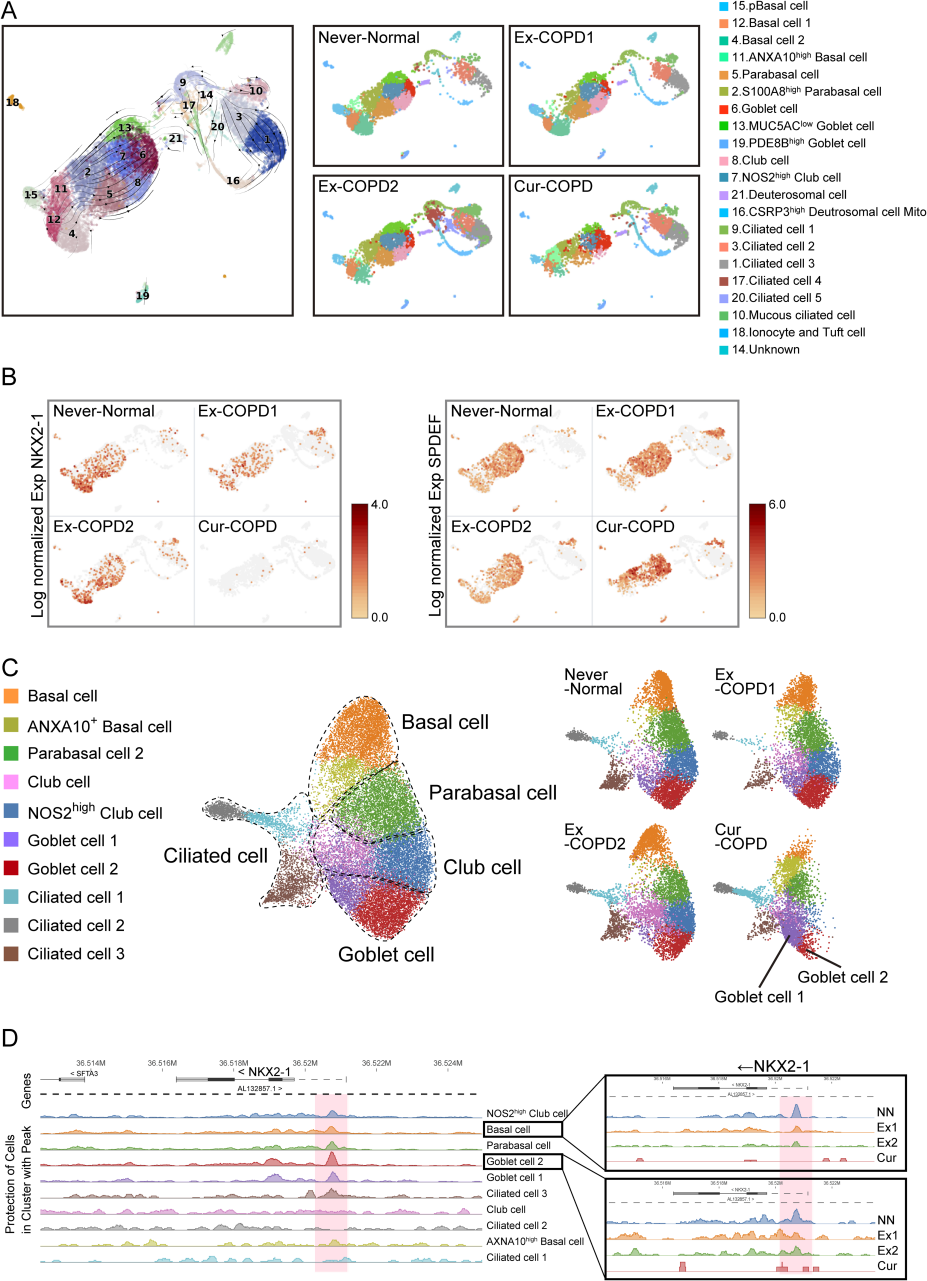
Integrating single‐cell RNA and ATAC **sequencing** to identify transcription factors associated with goblet cell differentiation in primary bronchial epithelial cells from a healthy never‐smoker, two ex‐smokers, and a current smoker with COPD. (A) RNA velocity analysis (left panel). Arrows indicate the predicted lineage trajectories. UMAP visualisation and cell cluster identification of scRNA‐seq dataset among groups (right panel). (B) Distribution of NKX2‐1 and SPDEF gene expression visualised by UMAP dimensionality reduction among groups. (C) UMAP visualisation and cell cluster identification of scATAC‐seq dataset among groups. (D) Chromatin accessibility profiles near NKX2‐1 across the cell clusters and groups [Correction added on 24 February 2026, after first online publication: Figure 6 has been corrected in this version.].

The dynamics of airway epithelial cells and their differentiation trajectory towards goblet cells were analysed by RNA velocity analysis (using scVelo). In never smokers with normal lung function, the cells differentiated from basal to goblet cells via the parabasal and club cells (Figure 6A and Figure S4). In contrast, the airway epithelia of ex‐smokers with COPD showed a decreased basal cell population and an increased pathway of differentiation from parabasal cells directly to goblet cells, bypassing club cells (Figure 6A and Figure S4). Moreover, the airway epithelium of current smokers with COPD exhibited an increased number of basal cells with high Annexin A10 expression that differentiated into parabasal cells, which subsequently differentiated into goblet cells while bypassing the club cell‐intermediate state (Figure 6A and Figure S4). The differentiation of airway epithelial cells into goblet cells is primarily governed by a regulatory axis. In this axis, the transcription factor SPDEF promotes goblet cell differentiation, while NKX2‐1, functioning as an upstream transcription factor, downregulates SPDEF expression. In the airway epithelium of ex‐smokers with COPD, compared to never smokers with normal lung function, *NKX2‐1* gene expression decreased in basal and parabasal cells and increased in mucous ciliated cells (Figure 6B). In current smokers with COPD, *NKX2‐1* expression decreased across all cell types (Figure 6B). Compared to never smokers with normal lung function, SPDEF expression was increased in mucous ciliated cells of ex‐smokers with COPD, whereas, in current smokers with COPD, it was elevated in S100A8‐high‐expressing parabasal cells, mucous ciliated, and goblet cells (Figure 6B). Using the chromatin accessibility data from scATAC‐seq, we performed cell cluster classification (Figure 6C). In the airway epithelium of current smokers with COPD, compared to never smokers with normal lung function, similar to the scRNA‐seq findings, we observed decreased populations of basal, parabasal, and club cells, along with an increase in annexin A10‐positive basal cells (Figure 6C). The chromatin accessibility of the transcription starting sites (TSS) of NKX2‐1 exhibited highly open chromatin states in basal, parabasal, and goblet cells (Figure 6D). Cell‐type‐specific comparisons among groups revealed that in basal and goblet cells, never smokers with normal lung function maintained open chromatin states conducive to *NKX2‐1* gene expression, whereas ex‐smokers and current smokers with COPD showed partially closed and closed chromatin states, respectively (Figure 6D). In never smokers with normal lung function, NKX2‐1 chromatin accessibility in parabasal and club cells does not reach the levels observed in goblet and basal cells, yet a similar trend across the three groups is still evident, although less pronounced. In current smokers with COPD, NKX2‐1 chromatin in parabasal and club cells consistently remained inaccessible, a finding that aligns with the reduced NKX2‐1 gene expression observed across all cell types in the scRNA‐seq analysis (data not shown).

## 4 Discussion

Cigarette smoke contributes to COPD pathogenesis through mucous exudates accumulation and inflammatory immune cells infiltration into subepithelial airway epithelium [23, 24]. Although smoking cessation improves respiratory symptoms and reduces lung function decline [25, 26], ex‐smokers with COPD remain susceptible to exacerbations and persistent airway inflammation similar to current smokers with COPD [27, 28, 29, 30]. However, the mechanisms underlying this sustained inflammatory state remain unclear.

This study provides novel mechanistic insights by demonstrating that, for more than 10 years after smoking cessation, airway epithelial barrier dysfunction persists in patients with COPD. Using primary bronchial epithelial cells from never smokers with normal lung function and ex‐smokers and current smokers with COPD, we identified three key findings. First, genes associated with ciliary formation and motility were progressively downregulated from never smokers to ex‐smokers to current smokers with COPD, which indicates persistent impairment of mucociliary clearance. Second, the expression of junction‐associated molecules remained low in both ex‐smokers and current smokers with COPD, suggesting irreversible disruption of epithelial barrier integrity. Third, using single‐cell RNA and ATAC sequencing, we identified distinct alterations in cell differentiation trajectories and population distributions, particularly in the persistence of goblet cell hyperplasia, which is mechanistically linked to epigenetic changes in the NKX2‐1 regulatory regions. Mucociliary clearance is a critical defence mechanism against microbial infection, and viral infections are also known to impair mucociliary function and disrupt tight junctions, thereby increasing bacterial transmigration [31, 32]. Our findings demonstrate that smoking‐induced changes in airway epithelial cell differentiation and barrier function are not fully reversible upon smoking cessation, which potentially explains the continued susceptibility to exacerbations in ex‐smokers with COPD. Furthermore, our results suggest that therapeutic strategies targeting epithelial barrier restoration and the regulation of cell differentiation through epigenetic modifications might be beneficial, even in ex‐smokers with COPD.

SPDEF expression in airway epithelium induces goblet cell differentiation and inflammation, with studies showing it triggers spontaneous eosinophilic inflammation and type 2 inflammatory mediator expression in mice [33]. Acting as a crucial regulatory counterbalance, NKX2‐1 suppressed SPDEF and type 2 chemokines expression in the airway epithelium, thereby controlling mucous cell metaplasia and inflammation [17]. Despite documented NKX2‐1 reduction in asthmatic airways and IL‐5‐positive nasal polyps, the precise mechanisms underlying allergen‐induced NKX2‐1 downregulation have yet to be elucidated [17, 34]. A similar knowledge gap exists regarding the molecular mechanisms behind these persistent effects, despite evidence that smoking‐related health risks can continue for decades after cessation. DNA methylation changes likely mediate many of these long‐term pathogenic effects of cigarette smoke exposure [35]. Interestingly, while cigarette smoke‐induced DNA methylation is potentially reversible, its recovery follows variable patterns after smoking cessation [36]. Research shows most methylated CpG sites return to never smoker levels within 5 years of quitting, yet specific gene methylation patterns remain altered for decades, explaining potential long‐term health consequences of previous smoke exposure [36, 37]. However, most studies examining cigarette smoke‐induced epigenetic changes have used peripheral blood samples rather than tissue‐specific analyses. Here, we demonstrated for the first time that, in small airway epithelial cells of patients with COPD, cigarette smoke‐induced epigenomic regulation leads to aberrant cell differentiation and disease progression. These epigenetic alterations persisted even after long‐term smoking cessation, resulting in sustained abnormal differentiation and decreased expression of epithelial barrier‐associated molecules.

This study has some limitations. Although we demonstrated persistent epigenetic changes in small airway epithelial cells from patients with COPD, our analysis was cross‐sectional rather than longitudinal, which makes it difficult to establish direct causality. Our findings were based on samples from patients with mild‐to‐moderate COPD and may not be generalizable to severe COPD. There was a sex imbalance between groups, with more females in the never smokers' group and more males in the COPD group, making it difficult to exclude potential sex‐related effects on our findings. Recent studies have reported sexual dimorphism in airway epithelial transcriptomic responses to smoking, involving epigenetic regulatory mechanisms such as differential transcription factor binding site enrichment, including oestrogen‐related receptor alpha [38]. The sample size for single‐cell analysis was limited, which may affect the statistical power and generalizability of our cellular differentiation findings. Although we focused on NKX2‐1‐mediated pathways, other epigenetic modifications and transcription factors may also contribute to the persistent changes in airway epithelial function after smoking cessation. Moreover, our single‐cell analyses revealed that the residual effects of smoking on epigenomic changes and subsequent gene expression alterations after smoking cessation may exhibit substantial inter‐individual variability. Further investigation with larger cohorts will be necessary to better define the magnitude and determinants of this variability in the reversibility of smoking‐induced epigenetic modifications.

In conclusion, epigenetic dysregulation of the NKX2‐1 locus in the COPD airway epithelium contributes to long‐lasting changes in cellular differentiation and barrier dysfunction, which remain evident despite smoking cessation. These findings provide a mechanistic explanation for the continued susceptibility to exacerbations in ex‐smokers with COPD and suggest that therapeutic strategies targeting epithelial barrier restoration and cell differentiation pathways may be beneficial, even in patients who quit smoking. Our results underscore the importance of examining epigenetic changes in airway epithelial cells to better understand the long‐term effects of cigarette smoke exposure on COPD pathogenesis.

## Author Contributions

**Ayaka Shiota:** investigation, validation, formal analysis. **Keiko Kan‐o:** conceptualization, methodology, data curation, supervision, funding acquisition, visualization, project administration, resources, writing – original draft, investigation, validation, formal analysis, software. **Yumiko Ishii:** methodology, data curation, validation, investigation, formal analysis, visualization. **Tomoaki Koga:** methodology, investigation, validation, formal analysis, writing – original draft, software. **Takeshi Sawada:** investigation, validation, formal analysis. **Kei‐ichiro Yasunaga:** investigation, validation, formal analysis. **Shingo Usuki:** investigation, validation, formal analysis. **Tatsuya Katsuno:** investigation, methodology, validation, formal analysis, writing – original draft, visualization. **Shigesato Inoue:** investigation, validation, formal analysis. **Tomohiro Ogawa:** investigation, validation, formal analysis. **Akihiro Jo:** investigation, validation, formal analysis. **Satoru Fukuyama:** conceptualization, supervision, resources. **Mitsuyoshi Nakao:** resources, supervision, software. **Hiroaki Ogata:** resources, data curation. **Mizuho A. Kido:** investigation, validation, visualization, formal analysis, software. **Sachiko Tsukita:** conceptualization, supervision, resources. **Koichiro Matsumoto:** conceptualization, supervision, resources. **Isamu Okamoto:** resources, supervision.

## Funding

This work was supported by JSPS KAKENHI, Grant Number JP24K11316; the Program of the Joint Usage/Research Center for Developmental Medicine; the Research Program for Inter‐University Research Network for High Depth Omics, IMEG, Kumamoto University; and the NEXT Promotion of Development of a Joint Usage/Research System Project: Coalition of Universities for Research Excellence Program (CURE), Grant No. JPMXP1323015486.

## Ethics Statement

This study involves human participants. The study protocol was approved by the Kyushu University Institutional Review Board for Clinical Research (2023‐44), and all participants provided written informed consent.

## Conflicts of Interest

The authors declare no conflicts of interest.

## Supporting information


**Data S1:** Supporting Information.

## Data Availability

Bulk RNA‐seq, bulk ATAC‐seq, scRNA‐seq, and scATAC‐seq data have been deposited in the Gene Expression Omunibus (GEO) under accession numbers GSE301910 (bulk RNA‐seq), GSE302115 (bulk ATAC‐seq), GSE302794 (scRNA‐seq), and GSE302411 (scATAC‐seq). Any additional data supporting this study's findings are available from the corresponding author upon reasonable request.
