# Supplementary material for: Epigenetic Dysregulation of the NKX2‐1/SPDEF Axis Drives Persistent Goblet Cell Differentiation and Epithelial Barrier Dysfunction in Chronic Obstructive Pulmonary Disease
Source: Respirology. 2026 Jan 20;31(5):484–97. doi: 10.1002/resp.70201 (PMC13125390; doi:10.1002/resp.70201)
Supplement: Supplementary file 1 — Data S1: Supporting Information. [file RESP-31-484-s001.pdf]

Supplemental Material

Epigenetic dysregulation of the NKX2-1/SPDEF axis drives persistent goblet cell differentiation and epithelial barrier dysfunction in chronic obstructive pulmonary disease

Ayaka Shiota[†1,2], Keiko Kan-o[†1,3]*, Yumiko Ishii[1,4], Tomoaki Koga[5], Takeshi Sawada[4], Kei-ichiro Yasunaga[6], Shingo Usuki[6], Tatsuya Katsuno[7,8], Shigesato Inoue[1,9], Tomohiro Ogawa[1], Akihiro Jo[1], Satoru Fukuyama[1,9], Mitsuyoshi Nakao[5], Hiroaki Ogata[1], Mizuho A. Kido[4], Sachiko Tsukita[10,11], Koichiro Matsumoto[12], Isamu Okamoto[1]

[1] Department of Respiratory Medicine, Graduate School of Medical Sciences, Kyushu University, Fukuoka, Japan

[2] Division of Respiratory Medicine, National Hospital Organization Fukuoka National Hospital, Fukuoka, Japan

[3] Department of Respiratory Medicine, Tokyo Women's Medical University, Tokyo, Japan

[4] Division of Histology and Neuroanatomy, Department of Anatomy and Physiology, Faculty of Medicine, Saga University, Saga, Japan

[5] Department of Medical Cell Biology, Institute of Molecular Embryology and Genetics, Kumamoto University, Kumamoto, Japan

[6] Liaison Laboratory Research Promotion Center, IMEG, Kumamoto University, Kumamoto, Japan

[7] Center for Anatomical Studies, Graduate School of Medicine, Kyoto University, Sakyo-ku, Kyoto, Japan

[8] KOKORO-Biology Group, Graduate School of Frontier Biosciences, Osaka University, Osaka, Japan

[9] Department of Respiratory Medicine, National Hospital Organization Omuta National Hospital, Omuta, Japan

[10] Advanced Comprehensive Research Organization, Teikyo University, Tokyo, Japan

[11] Laboratory of Barriology and Cell Biology, Graduate School of Frontier Biosciences, Osaka University, Osaka, Tokyo

[12] Department of Medicine, Division of Oral and Medical Management, Fukuoka Dental College, Fukuoka, Japan

[†]These authors contributed equally to this work.

*** Corresponding author**

Keiko Kan-o, MD, PhD

Department of Respiratory Medicine, Tokyo Women's Medical University, Tokyo, Japan

8-1 Kawadacho, Shinjuku-ku, Tokyo 162-8666, Japan

Tel: +81-3-3353-8111

E-mail: kano.keiko@twmu.ac.jp

Supplementary Methods

Culture of primary bronchial epithelial cells

Primary bronchial epithelial cells (PBECs) were cultured in flasks coated with collagen (Cell Applications, Inc., San Diego, CA, USA) containing supplemented bronchial epithelial growth medium (BEGM; Lonza, Basel, Swiss) at 37°C in 5% CO_2 and used for the air-liquid interface (ALI) culture within four passages. For ALI culture, cells were seeded onto collagen-coated Transwell inserts (0.33-cm^2 Polyethylene terephthalate, 0.4-µm pore size; Corning, Glendale, AZ, USA) at a density of 2.4×10^5 cells/cm^2 with 200 µL apical volume and 500 µL basal volume of BEGM. After 48–72 h, the apical medium was removed, and the cells were maintained with 500 µl of PneumaCult™-ALI Maintenance Medium (STEMCELL technologies, Tokyo, Japan) in the basal chamber. The medium was renewed every other day and the monolayers were allowed to differentiate under ALI conditions for 21 days. For comparison among never-smoker with normal lung function, ex-smokers with COPD, and current smokers with COPD, cells derived from at least one sample from each group were differentiated at the same time and used for experiments. Table S1 shows the demographic data of the patients from whom the cells used in the corresponding experiments in each figure were derived.

Electron microscopy

Transmission Electron Microscopy (TEM)

The cultured cells were fixed with 4% paraformaldehyde and 2.5% glutaraldehyde in 0.1 M phosphate buffer at 4˚C overnight. The specimens were post-fixed with ferrocyanide-reduced osmium tetroxide {1% [w/v] OsO_4}. After fixation, the tissues were dehydrated in a graded series of ethanol solutions (50%, 60%, 70%, 80%, 90%, 99%, and 100%) and embedded in epoxy resin according to a standard procedure. Ultrathin sections were prepared by using an ultramicrotome (UC7; Leica Microsystems, Solms, Germany). Sections were stained with uranyl acetate and lead citrate and observed under an electron microscope (JEM-1400flash, JEOL, Tokyo. Japan).

Backscattered electron scanning electron microscopy (SEM)

The samples were fixed, treated, embedded, and sectioned using an ultramicrotome (ARTOS 3D, Leica Microsystems), as described above, with a diamond knife (SYM Jumbo, 45°, SYNTEK) to obtain 70–90 nm serial sections. Serial sections were collected for BSE-SEM using a cleaned silicon wafer strip held by a micromanipulator (MN-153; NARISHIGE, Tokyo, Japan). Sections were stained at room temperature with 2% (w/v) aqueous uranyl acetate (20 min) and Reynolds lead citrate (3 min). Sections were imaged using a scanning

electron microscope (SEM; JSM-7900F, JEOL).

RNA sequencing

Total RNA from PBECs cultured under ALI conditions from never-smoker with normal lung function (n=3), ex-smoker with COPD (n=2), and current smokers with COPD (n=3) was extracted using TRIzol reagent (Thermo Fisher Scientific, Waltham, MA, USA. Poly-A RNA was isolated using the NEBNext Poly(A) mRNA Magnetic Isolation Module {New England BioLab (NEB), Ipswitch, MA, US}. For sequencing, a cDNA library was synthesized using a NEBNext Ultra II RNA Library Prep Kit (Illumina, San Diego, CA, USA). Sequencing was performed on a NextSeq 500 sequencer (Illumina) with 75-bp single-end reads. The resulting reads were mapped to the University of California (UCSC, Santa Cruz, CA, USA) hg19 reference genome using a spliced transcript alignment to a reference (STAR) Aligner. The number of reads was calculated using RNA sequencing (RNA-seq) by Expectation Maximization (RSEM), and differentially expressed genes (DEGs) among samples were analyzed by RNAseqChef.[1]

Assay for Transposase-Accessible Chromatin via Sequencing

ALI-PBECs from never-smoker with normal lung function (n=3), ex-smoker with COPD

(n=2), and current smokers with COPD (n=3) were dissociated into single cells and subjected to transposase-Accessible Chromatin via Sequencing (ATAC-seq) using an ATAC-seq kit (Active Motif, #53150) according to the manufacturer's instructions.[2] Briefly, 10k single cells were centrifuged at 500 xg for 5 min (4℃), and 1,000 xg for 5 min (4℃) and washed with 100 μL of ice-cold PBS. Cells were subsequently lysed using ice-cold ATAC lysis buffer and immediately spun at 500 xg for 10 min (4℃). The pellet was resuspended in the transposase reaction mix for the Tn5 transposase tagmentation for 30 min at 37℃ with shaking at 800 rpm, and the sample was purified using DNA purification column. Eluted DNA was next indexed and amplified using PCR (72℃ 5 min, 98℃ 30 sec, 12 cycles of 98℃ 10 sec, 63℃ 30 sec, 72℃ 1 min, and Hold at 10℃), and then DNA was purified using SPRI select beads (Beckman Coulter, Brea, CA, USA). The sample quality was assessed using a high-sensitivity DNA D1000 strip and TapeStation (Agilent, Santa Clara, CA, USA). DNA libraries were multiplexed and sequenced on NextSeq500 (Illumina). For data analysis, quality and adapter filtering were applied to raw reads using Trim Galore before alignment to the human assembly hg19 using bowtie2. The Picard MarkDuplicate tool was used to remove reads with the same start sites and orientations. The BEDTools suite was used to create the read-density profiles. Enriched regions were identified using MACS2 and scored against matched input libraries. DiffBind was used to classify differentially accessible regions (DARs) between the

two conditions using fold change > 1.5 and FDR-adjusted p-value < 0.05. Peak gene associations were made using the linear genomic distance to the nearest transcription start site using Homer.

Quantitative reverse-transcription PCR

Total RNA was isolated from PBECs using the TRI Reagent (Molecular Research Centre, Inc., Cincinnati, OH, USA). Reverse transcription was performed using Multiscribe Reverse Transcriptase (Invitrogen, Waltham, MA, USA). Real-time quantitative RT-PCR was performed once per sample using TB Green Premix Ex Taq™ II (Takara Bio Inc., Shiga, Japan) and a Thermal Cycler Dice Real-Time System II (Takara Bio Inc.). The mRNA expression levels were calculated from the threshold cycle according to the ΔΔCt method. Target gene expression levels were normalized to those of GAPDH. Primer sequences are provided in Table S2.

Immunofluorescence staining

The differentiated cells were washed twice with PBS and fixed with cold methanol for 10 min. After blocking with PBS containing 1% bovine serum albumin (BSA) for 30 min at room temperature, the cells were incubated with each primary antibody prepared in PBS containing

1% BSA at 4 °C overnight, followed by incubation with Alexa Fluor 488-conjugated goat anti-rabbit IgG antibody (diluted 1:500; Abcam, Cambridge, UK, #150077) or Alexa Fluor 488-conjugated goat anti-rat IgG antibody (diluted 1:500; Thermo Fisher Scientific, #11006) and Alexa Fluor 568-conjugated goat anti-mouse IgG antibody (diluted 1:500; Abcam, #175473) for 2 h at room temperature. Then F-atcin and nuclear were stained with Phalloidin-iFlour 647 Reagent (diluted 1:1000; Abcam, #176759) and 4', 6-diamidino-2-phenylindole (DAPI) (diluted 1:1000; FUJIFILM Wako Chemical Corporation, Osaka, Japan, #340-07971), respectively. The primary antibodies were as follows: rabbit anti-claudin-3 polyclonal antibody (diluted 1:200; Thermo Fisher Scientific, #34-1700); rabbit anti-occludin polyclonal antibody (diluted 1:200; Thermo Fisher Scientific, #71-1500); mouse anti-E-cadherin monoclonal antibody (diluted 1:500; Takara bio inc, Shiga, Japan, #M108); and mouse anti-MUC5AC monoclonal antibody (diluted 1:500; Thermo Fisher Scientific, #MA5-12178).

Fluorescence images were obtained using a Zeiss LSM 700 confocal microscope (Carl Zeiss, Oberkochen, Germany) with a 40× oil 1.4 numerical aperture objective lens. Z-stack images were captured by taking an average of 20 slices at 1-μm intervals, ranging from the bottom, where the nucleus is visible, to the top, where F-actin or MUC5AC can be observed. For subsequent quantitative analysis of the fluorescence intensity of junction-associated proteins

or calculation of the percentage of goblet cells, Z-stack images were randomly captured from a single sample in three fields. Images were processed using ZEN3.5 software (blue edition; Carl Zeiss).

For quantitative analysis of the fluorescence intensity of junction-associated proteins, the values obtained by multiplying the fluorescence intensity (50–200) and the pixel count of junction-associated proteins were summed in all imaging slices of the Z-stack where the expression of junction-associated proteins was observed. The average of the values from three fields within each sample was then calculated. The average intensity values of the controls were set at 100%, and the relative intensity ratios of samples from current and ex-smokers with COPD were calculated. To analyze the analysis process of Z-stack fluorescence microscopy images for counting goblet cells, we used ImageJ/FIJI.[3] To count the number of nuclei and MUC5AC positive cells in the field, we applied the images to two plugins: the 3D segmentation plugin (Tools for Analysis of Nuclear Genome Organization, TANGO) and the 3D Objects Counter Plugin.[4, 5] Nuclei were segmented using Otsu's thresholding method.[6] MUC5AC-positive cells were selected under the cell apex using a 3D Objects Counter Plugin to avoid extraction of the mucus layer above the cells. Distribution of MUC5AC-positive cells in the samples was heterogeneous. Therefore, the correlations between the average intensity

of junction-associated proteins and the percentage of MUC5AC-positive cells from three random fields within a sample are displayed in the correlation graphs.

For some experiments, immunofluorescent images were obtained using a Andor BC43 Benchtop confocal microscope (Andor, Belfast, UK), LSM800 equipped with Airyscan (Carl Zeiss). Andor BC43 Image stacks were obtained at 2,048 × 2,000 pixel resolution using a Nikon 20× 0.25 NA and 60x 1.4 NA oil immersion objectives. BC43 Images were acquired with the Fusion software (Oxford Instruments) and processed with the Imaris software (Oxford Instruments). LSM 800 confocal microscope with a Plan Apochoromat 63×/1.40 NA oil DIC M27 objective lens and an LSM 800 confocal unit with Airyscan module (Carl Zeiss). Z-stack images were captured by taking an optimal slice of Airyscan unit at 0.13µm intervals. The images were Airyscan processed automatically with high level using the Zeiss Zen Blue 3.5 software package (blue edition; Carl Zeiss).

Preparation and treatment of cigarette smoke extracts (CSE)

CSE was prepared by combusting two cigarettes (Marboro) and bubbling mainstream cigarette smoke into 20 mL cell culture medium. The CSE was sterile-filtered through a 0.22-µm filter (33-mm Millex GV; Merck Millipore, Billerica, MA) after adjustment of the

pH to 7.4 and considered as 100% CSE. This preparation was standardized by measuring the absorbance at wavelength 320 nm, freshly prepared for each experiment.

Measurement of transepithelial electrical resistance

Transepithelial electrical resistance (TEER) was evaluated using a Millicell-ERS 2 V-Ohm meter (Millipore Co., Bedford, MA). If the apical chamber did not contain any medium, the cell culture medium was added into the apical chamber and incubated for 20 minutes at 37°C in 5% CO_2 before TEER measurement. The electrode was soaked in 70% ethanol and rinsed with the culture medium before use. TEER was calculated by the following equation [7]: TEER (Ωcm^2)=(Rsample – Rblank) × effective membrane area (cm^2).

Single-cell RNA sequencing

The differentiated cells were then dissociated using TrypLE (Thermo Fisher Scientific, Waltham, MA, USA) for 15 min at 37°C, washed twice with PBS (-), and resuspended in 0.04% BSA/PBS. The dataset contained 4,122 cells and 6,304 genes per cell (average of four samples). Gene expression was visualized using Loupe Browser software (10× Genomics, Pleasanton, CA, USA). Twenty-one clusters were visualized using specific marker genes including 2 basal cells; TP63+ KRT5+ SCGB1A1-, proliferating basal cell (pBasal); TP63+

KRT5+ SCGB1A1- MKI67+, ANXA10^{high} basal cell; TP63+ KRT5+ SCGB1A1- ANXA10+, 2 parabasal cell; TP63- KRT5+ SCGB1A1- S100A8+/-, 2 Club cell; TP63- KRT5- SCGB1A1+ NOS2+/-, 5 Ciliated cell; FOXJ1+ DEUP1- FOXN4-, Mucous Ciliated cell; FOXJ1+ DEUP1- FOXN4- MUC5AC/5B+, 2 Deuterosomal cell; FOXJ1+ DEUP1- FOXN4+, and 3 Goblet cell; MUC5AC+ MUC5B+/- PDE8B+/-, Ionocyte & Tuft cell; CFTR+ FOXI1+. Differentially expressed genes, such as CLDNs, were visualized as heat maps. To study the cellular dynamics, RNA velocity was analyzed using the scVelo pipeline.[8]

Single-cell ATAC sequencing

The datasets were first analyzed using the Cell Ranger ATAC software (10x Genomics) and visualized using UMAP. The dataset contained 5610 cells and 4847 high-quality fragments per cell (averaged over four samples). Visualization of accessible regions was performed using the Loupe Browser software. Ten clusters were visualized using gene-specific chromatin accessibility: basal cell; TP63 KRT5, ANXA10^{high} basal cell; TP63 KRT5 ANXA10, parabasal cells; TP63, 2 Club cell; SCGB1A1 NOS2, 2 Goblet cell; MUC5AC, and 3 Ciliated cells; FOXJ1 CWH43.

References

1. Etoh K, Nakao M. A web-based integrative transcriptome analysis, RNAseqChef, uncovers the cell/tissue type-dependent action of sulforaphane. J Biol Chem. 2023;299:104810.

2. Buenrostro JD, Giresi PG, Zaba LC, Chang HY, Greenleaf WJ. Transposition of native chromatin for fast and sensitive epigenomic profiling of open chromatin, DNA-binding proteins and nucleosome position. Nat Methods. 2013;10:1213-8.

3. Schneider CA, Rasband WS, Eliceiri KW. NIH Image to ImageJ: 25 years of image analysis. Nat Methods. 2012;9:671-5.

4. Ollion J, Cochennec J, Loll F, Escude C, Boudier T. TANGO: a generic tool for high-throughput 3D image analysis for studying nuclear organization. Bioinformatics. 2013;29:1840-1.

5. Bolte S, Cordelieres FP. A guided tour into subcellular colocalization analysis in light microscopy. J Microsc. 2006;224:213-32.

6. Xing J, Yang P, Qingge L. Robust 2D Otsu's Algorithm for Uneven Illumination Image Segmentation. Comput Intell Neurosci. 2020;2020:5047976.

7. Sekiyama A, Gon Y, Terakado M, Takeshita I, Kozu Y, Maruoka S, et al. Glucocorticoids enhance airway epithelial barrier integrity. Int Immunopharmacol. 2012;12:350-7.

8. Bergen V, Lange M, Peidli S, Wolf FA, Theis FJ. Generalizing RNA velocity to transient

cell states through dynamical modeling. Nat Biotechnol. 2020;38:1408-14.

Table S1

Patients' demographic data

Figure 1 (Bulk RNA-seq, Bulk ATAC-seq)			
	Never smokers with normal lung function (n=3)	Ex-COPD (n=2)	Current COPD (n=3)
Age, years [a]	73/67/73	74/71	72/68/77
Sex	Male/Female/Female	Male/Male	Female/Male/Male
Smoking history, pack-years	0/0	40/30	40/48/47
Smoking cessation period, years	–	14/21	–
FEV_1 % predicted (post-BD), %	103/110/114	90/74	77/102/78
FEV_1/FVC (post-BD), % [a]	74/82/72	64/60	60/65/66
Treatment, n (%)			
None	4 (100.0)	2 (100.0)	2 (66.7)
LAMA/LABA	0 (0.0)	0 (0.0)	1 (33.3)

Figure 2 (qPCR)			
	Never smokers with normal lung function (n=8)	Ex-COPD (n=10)	Current COPD (n=9)
Age, years [a]	72.5 (67.0-80.5)	73.5 (66.5-74.3)	73.0 (68.5-76.5)
Male sex, n (%)	1 (12.5)	9 (90.0)	8 (88.9)
Smoking history, pack-years [a]	1.25 [‡]	40.0 (30.0-49.5)	41.0 (28.0-55.0)
Smoking cessation period, years [a]	42.0[‡]	15.5 (12.25-26.0)	–
FEV_1 % predicted (post-BD), % [a]	112.5 (97.0-123.5)	89.5 (75.8-97.5)	77.0 (56.5-93.5)
FEV_1/FVC (post-BD), % [a]	77.0 (74.5-78.8)	63.0 (57.5-65.5)	60.0 (43.0-64.5)
Treatment, n (%)			
None	8 (100.0)	9 (90.0)	5 (55.6)
LAMA/LABA	0 (0.0)	0 (0.0)	3 (33.3)
LAMA/LABA/ICS	0 (0.0)	1 (10.0)	1 (11.1)

Figure 3 (IF)			
	Never smokers with normal lung function	Ex-COPD (n=6)	Current COPD (n=6)

	(n=4)		
Age, years [a]	69.5 (67.0-72.8)	70.0 (62.8-75.5)	73.0 (67.8-76.3)
Male sex, n (%)	1 (25.0)	5 (83.3)	5 (83.3)
Smoking history, pack-years [a]	0.0	42.5 (24.0-73.5)	41.0 (28.5-67.3)
Smoking cessation period, years [a]	−	15.5 (10.0-27.5)	−
FEV_1 % predicted (post-BD), % [a]	102.0 (96.5-118.8)	88.0 (59.8-101.8)	74.5 (61.3-98.0)
FEV_1/FVC (post-BD), % [a]	78.0 (75.0-79.5)	62.0 (43.8-67.5)	58.5 (44.0-64.5)
Treatment, n (%)			
None	4 (100.0)	5 (83.3)	2 (33.3)
LAMA/LABA	0 (0.0)	0 (0.0)	3 (50.0)
LAMA/LABA/ICS	0 (0.0)	1 (16.7)	1 (16.7)

Figure 4 (IF)			
	Never smokers with normal lung function (n=4)	Ex-COPD (n=6)	Current COPD (n=8)
Age, years [a]	69.5 (67.0-72.8)	70.0 (62.8-75.5)	70.5 (65.0-75.5)
Male sex, n (%)	1 (25.0)	5 (83.3)	7 (87.5)
Smoking history, pack-years [a]	0.0	42.5 (24.0-73.5)	34.5 (27.0-53.3)
Smoking cessation period, years [a]	−	15.5 (10.0-27.5)	−
FEV_1 % predicted (post-BD), % [a]	102.0 (96.5-118.8)	88.0 (59.8-101.8)	74.5 (66.8-95.5)
FEV_1/FVC (post-BD), % [a]	78.0 (75.0-79.5)	62.0 (43.8-67.5)	62.0 (48.0-65.0)
Treatment, n (%)			
None	4 (100.0)	5 (83.3)	4 (50.0)
LAMA/LABA	0 (0.0)	0 (0.0)	3 (37.5)
LAMA/LABA/ICS	0 (0.0)	1 (16.7)	1 (12.5)

Figure 5 (CSE exposure)	
	Never smokers with normal lung function (n=3)
Age, years [a]	67.0 (35.0-69.0)
Male sex, n (%)	1 (33.3)
Smoking history, pack-years [a]	−
Smoking cessation period, years [a]	−
FEV_1 % predicted (post-BD), % [a]	110.0 (104.0-118.0)
FEV_1/FVC (post-BD), % [a]	82.0 (80.0-84.0)

Treatment, n (%)	
None	4 (100.0)

Figure 6, 7 (scRNA-seq, scATAC-seq)			
	Never smokers with normal lung function (n=1)	Ex-COPD (n=2)	Current COPD (n=1)
Age, years	67	80/71	72
Sex	Female	Female/Male	Female
Smoking history, pack-years	0	6/30	40
Smoking cessation period, years	–	38/21	–
FEV_1 % predicted (post-BD), %	101	110/74	77
FEV_1/FVC (post-BD), % [a]	80	60/60	60
Treatment, n (%)			
None	1 (100.0)	2 (100.0)	0 (0.0)
LAMA/LABA	0 (0.0)	0 (0.0)	1 (100.0)

Supplementary Figure 2 (TEER measurement)			
	Never smokers with normal lung function (n=4)	Ex-COPD (n=4)	Current-COPD (n=4)
Age, years [a]	68.0 (43.0-72.0)	71.5 (65.5-73.8)	70.5 (55.3-76.0)
Male sex, n (%)	1 (25.0)	3 (75.0)	4 (100.0)
Smoking history, pack-years [a]	0.6 (0.0-2.6) [†]	41.5 (40.0-48.3)	27.8 (24.8-38.1)
Smoking cessation period, years [a]	31.0 (20.0-42.0) [†]	11.5 (10.0-13.8)	–
FEV_1 % predicted (post-BD), % [a]	108.0 (87.0-123.8)	90.0 (72.5-98.5)	70.5 (47.3-86.3)
FEV_1/FVC (post-BD), % [a]	75.0 (72.5-79.8)	65.5 (61.0-69.0)	65.0 (41.0-65.0)
Treatment, n (%)			
None	4 (100.0)	2 (50.0)	2 (50.0)
LABA	0 (0.0)	1 (25.0)	0 (0.0)
LAMA/LABA	0 (0.0)	1 (25.0)	1 (25.0)
LAMA/LABA/ICS	0 (0.0)	0 (0.0)	1 (25.0)

Data are presented as the medians (interquartile range) [a].

[†] The percentage of former smokers is 50% (two of four), and the values shown as median values (interquartile range) of smoking history and smoking cessation period are calculated in only former smokers.

‡ The percentage of former smokers is 12.5% (one of eight), and the values shown as median values (interquartile range) of smoking history and smoking cessation period are calculated in only former smokers. FEV_1, forced expiratory volume in one second; FVC, forced vital capacity; BD, bronchodilator; LABA, long-acting β_2-agonist; LAMA, long-acting muscarinic antagonist; ICS, inhaled corticosteroids.

Table S2

Primer sequences used for qRT-PCR.

CLDN1	Sense: 5′ CCGGCGACAACATCGTGAC 3′
	Antisense: 5′ CGGGTTGCTTGCAATGTGC 3′
CLDN2	Sense: 5′ TTATGTCGGTGCCAGCATTG 3′
	Antisense: 5′ CACACTGGGTGATGCCTGTG 3′
CLDN3	Sense: 5′ CGCGAGAAGAAGAAGTACACGG 3′
	Antisense: 5′ CCTTAGACGTAGTCCTTGCGG 3′
CLDN4	Sense: 5′ ATCGGCAGCAACATTGTCAC 3′
	Antisense: 5′ GCGAGTCGTACACCTTGCAC 3′
CLDN7	Sense: 5′ GGCTTCCTGGCCATGTTTG 3′
	Antisense: 5′ GCAAGACCTGCCACGATGAAA 3′
CLDN8	Sense: 5′ AACTTCTGGGAAGGACTGTGGATG 3′
	Antisense: 5′ GGAGAAAGAGCCAGCAGGGAA 3′
CLDN10b	Sense: 5′ CACGGTCATCACAACCGCCA 3′
	Antisense: 5′ TGTATATAACCGTCCAGCGCCAG 3′
CLDN12	Sense: 5′ TCAAAGCATGAAGAAAACGAGGCA 3′
	Antisense: 5′ AAAGGACTGTGGCTGCGTGG 3′
CLDN15	Sense: 5′ AGGAAGCAGAGAGACCCACA 3
	Antisense: 5′ AGAACCCCTAGGGAACTGGA 3′
CLDN18.1	Sense: 5′ TGTCCACCACCACATGCCAAG 3′
	Antisense: 5′ ACGGGGTTGTCGTACAGGTC 3′
CLDN25	Sense: 5′ CAAGAGGCGGCTTGGTCTCC 3′
	Antisense: 5′ GGGCACCTCCAAACTCCCAC 3′
OCLN	Sense: 5′ TGCATGTTCGACCAATGC 3′
	Antisense: 5′ AAGCCACTTCCTCCATAAGG 3′
TJP1 (ZO-1)	Sense: 5′ AAGATGTCCGCCAGAGCTGC 3′
	Antisense: 5′ AGCGTCACTGTATGTTGTTCCC 3′
F11R (JAM-A)	Sense: 5′ TGCCTATAGCCGAGGCCACT 3′
	Antisense: 5′ATGATAGGCGGTGAGCCGAC 3′
E-Cad	Sense: 5′ GAAGGTGACAGAGCCTCTGGAT 3′
	Antisense: 5′ GATCGGTTACCGTGATCAAATC 3′
MUC5AC	Sense: 5′ TGCAGCTATGTGCTGACCAA 3′
	Antisense: 5′ GCTCAGTGTCACGCTCTTCA 3′
GAPDH	Sense: 5′ GCCACTAGGCGCTCACTGTTC 3′
	Antisense: 5′ GTGACCAGGCGCCCAATACG 3′

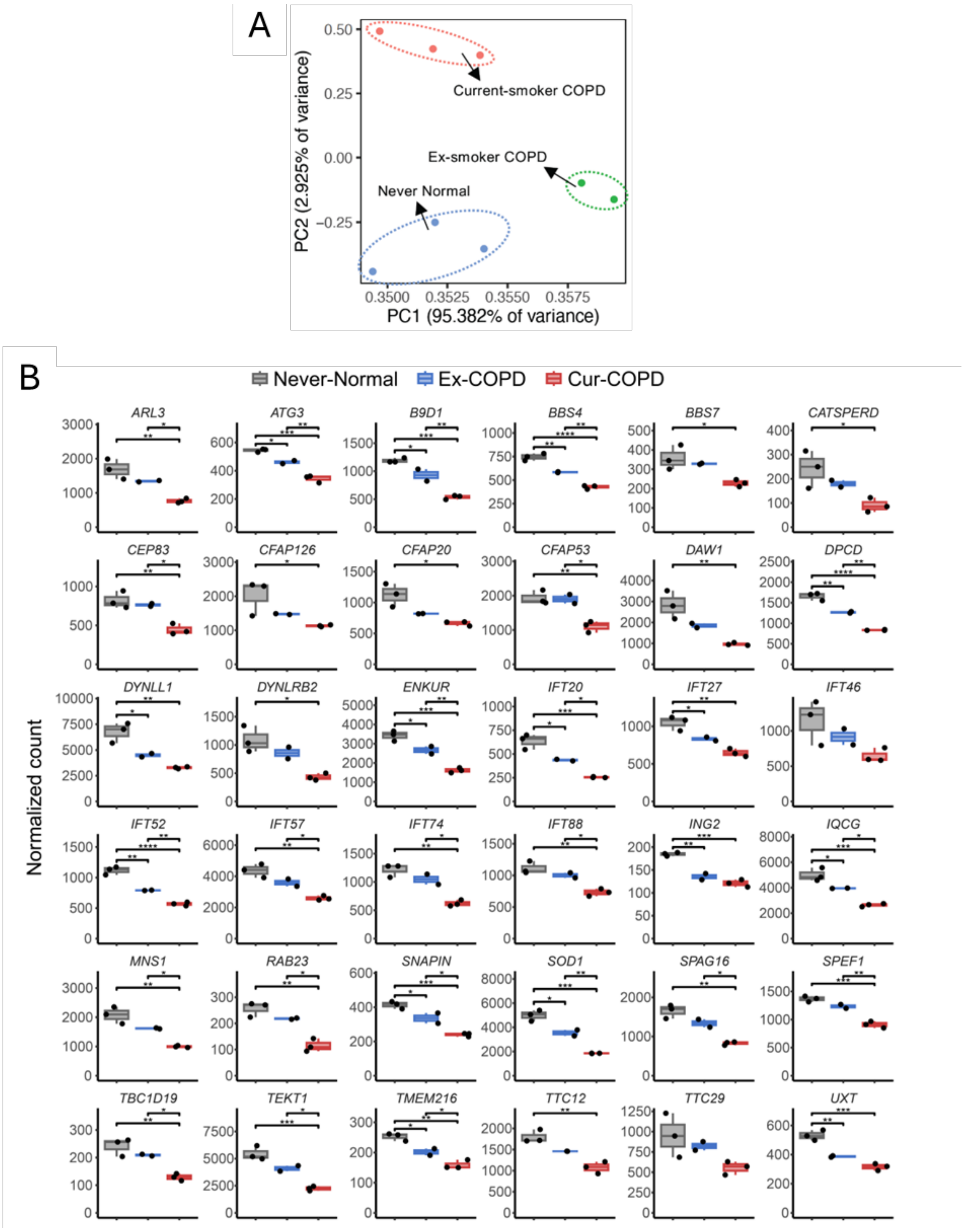

Figure S1. Bulk RNA sequencing analysis of differentiated primary bronchial epithelial cells from never-smokers with normal lung function (n=3), ex-smokers (n=2), and current smokers (n=3) with COPD.

(A) Principal Component Analysis (PCA) of the transcriptome from samples across the three groups. (B) Box plots comparing expression levels of genes related to cilium organization among groups. *p<0.05, **p<0.001, ***p<0.0001 by one-way ANOVA with *post hoc* with Tukey test.

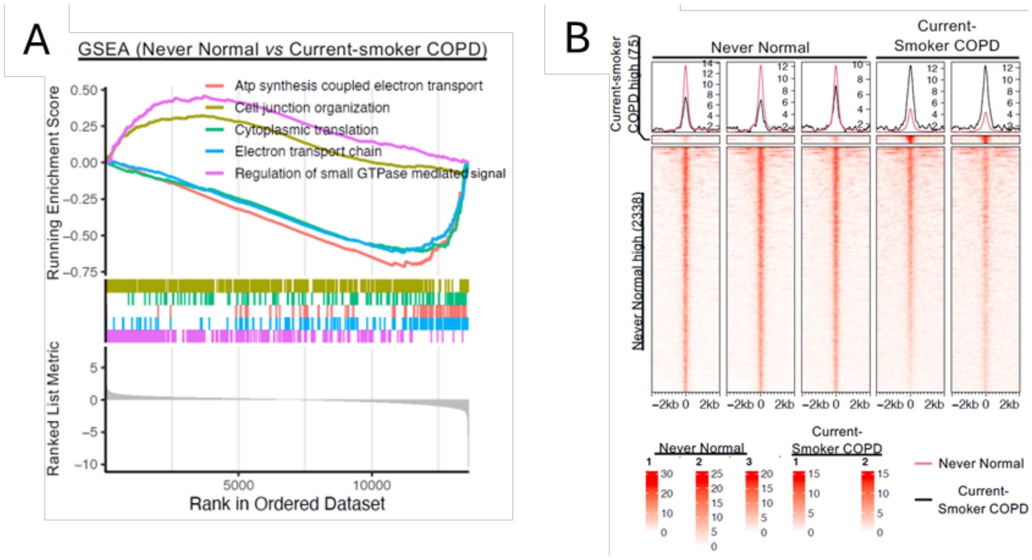

Figure S2. Bulk ATAC sequencing analysis of differentiated primary bronchial epithelial cells from never-smokers with normal lung function (n=3) and current smokers with COPD (n=3).

(A) Gene set enrichment analysis of the transcriptome from never-smokers with normal lung function and current smokers with COPD. (B) Heatmap of DARs from never-smokers with normal lung function and current smokers with COPD by ATAC-seq analysis.

*p<0.05, **p<0.001, ***p<0.0001 by one-way ANOVA *post hoc* with Tukey test.

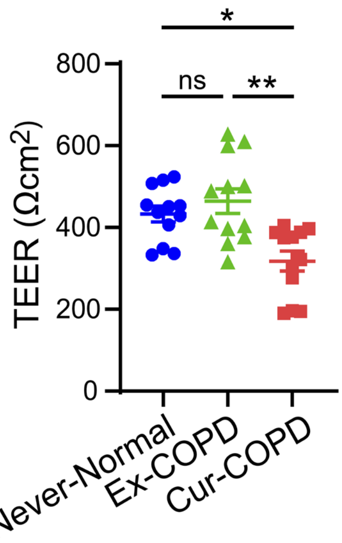

Figure S3. Analysis of transepithelial electrical resistance in primary bronchial epithelial cells differentiation from never-smokers with normal lung function, ex-smokers, and current smokers with COPD.

Cells from never-smokers with normal lung function, ex-smokers, and current smokers with COPD were cultured simultaneously under ALI conditions for 21 days and TEER was measured. Data represent the mean ± SEM (n=4 independent donors with three replicates per group). *p<0.01, **p<0.001 by one-way ANOVA *post hoc* with Tukey test.

TEER, transepithelial electrical resistance; ALI, ns, not significant.

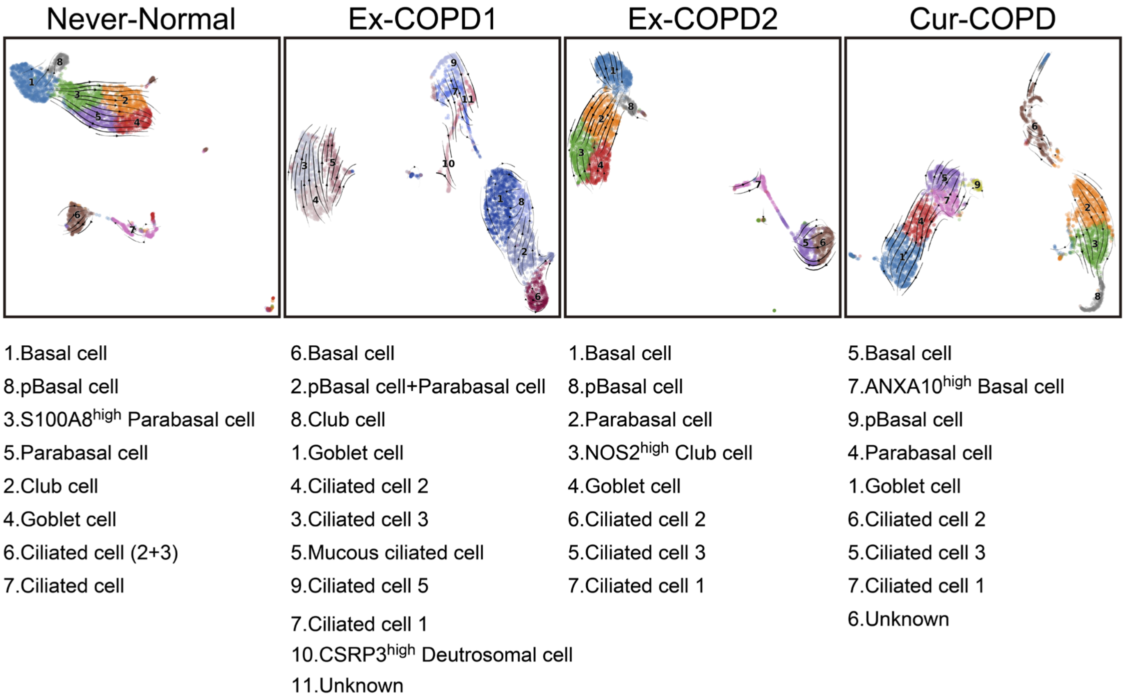

Figure S4. single-cell Velo analysis comparing differentiated primary airway epithelial cells from a never-smoker with a normal lung function as well as two ex-smokers, and a current smoker with COPD.

RNA velocity analysis using scVelo was used to visualize the predicted lineage trajectories in each group.
